# Sublingual and Buccal Delivery: A Historical and Scientific Prescriptive

**DOI:** 10.3390/pharmaceutics17081073

**Published:** 2025-08-20

**Authors:** Sina Bahraminejad, Hassan Almoazen

**Affiliations:** Department of Pharmaceutical Sciences, University of Tennessee Health Science Center, 881 Madison Ave, Rm 661, Memphis, TN 38018, USA

**Keywords:** sublingual drug delivery, buccal drug delivery, rapid onset absorption, dose limited absorption, penetration enhancement

## Abstract

In this review, our intention was to shed some light on the history of sublingual and buccal delivery over the past 75 years. By searching the query sublingual and buccal, we noticed four steady growth periods in the number of publications between 1950 and 2025. The early phase of sublingual and buccal drug delivery (1950–1982) saw limited attempts to explore this delivery route. The exploratory growth phase (1983–1993) was marked by the use of nitroglycerin to treat angina, calcium channel blockers to treat hypertension, ACE inhibitors to treat heart conditions, the use of opioids in pain management therapy, and peptide and hormonal therapy. The diversification and discovery phase (1994–2009) was marked by the introduction of small molecules for the treatment of opioid use disorder and analgesia, the use of animal models to enhance the pharmacokinetic understanding of the sublingual and buccal route, the use of penetration enhancers, peptide and hormonal therapy, and few marked FDA drug approvals in this area. The innovation and integration phase (2010–2025) was marked by the use of nanoparticles, multilayered mucoadhesive systems, pediatric formulations (fast-dissolving films and tablets), immunotherapy and vaccine delivery, and a broad spectrum of therapeutic agents, such as steroids, antifungals, cannabinoids, antidepressants, antipsychotics, and narcotics (e.g., buprenorphine and apomorphine), novel formulations of fentanyl and diazepam for pain and seizure control, and the introduction of buccal vitamin D3 sprays. Understanding the history of sublingual and buccal delivery demonstrates a growing area of research focused on enhancing mucosal drug delivery for achieving local and systemic therapeutic benefits.

## 1. Introduction

Oral administration has historically been the most prevalent and accepted drug delivery approach, favored for its convenience and non-invasive nature by both patients and clinicians. Traditionally, mucosal drug administration has been employed for localized effects; however, the oral cavity is now recognized as a viable route for systemic delivery. The observed shift is a result of the special anatomical and physiological features of the oral mucosa, specifically, its extensive vascularization and direct connection to systemic circulation allow certain drugs to avoid first-pass metabolism within the gastrointestinal tract and liver. Given these characteristics, oral mucosal administration—sublingual and buccal routes in particular—presents a compelling alternative to established enteral and parenteral approaches, especially for drugs necessitating rapid action or exhibiting instability within the gastrointestinal tract [1,2].

Although the oral epithelium isn’t highly permeable, the sublingual and buccal tissues have benefits like easy access, sustained drug release, and better patient compliance. Historically, fast-acting drugs like nitroglycerin, which are significantly broken down in the gut, have been given sublingually. The sublingual route’s acceptable permeability enables rapid drug absorption via passive diffusion across the lipoidal membrane, achieving onset times comparable to injections. The effectiveness of such routes is, however, greatly influenced by the physicochemical properties inherent to the drug (solubility, permeability, stability, hydrophilicity) and the design parameters of the delivery system. Advances in pharmaceutical sciences have led to the creation of mucoadhesive formulations designed to prolong mucosal residence time, optimize drug concentration gradients, and increase bioavailability [3,4]. This review analyzes the historical evolution and scientific principles underlying sublingual and buccal drug delivery systems, underscoring their expanding significance in modern pharmaceutical development and the urgent care of patients. PubMed served as the primary data source for this review, which tracked the annual publication count indexed between 1950 and 2025 using the search query “sublingual and buccal.” This bibliometric analysis incorporated a comprehensive review of relevant scientific literature and regulatory documents sourced from Scopus, Web of Science, ScienceDirect, and regulatory agencies including the FDA, EMA, and WHO. Inclusion criteria were limited to peer-reviewed publications and regulatory listing (orange book) pertaining to formulation technologies, therapeutic applications, and the anatomical characteristics of the oral mucosa.

### 1.1. The Early Phase of Sublingual and Buccal Drug Delivery (1950–1982)

The emergence of sublingual and buccal drug delivery during the 1950s–1980s started with the administration of steroids via buccal mucous membranes. Due to the inactivation of most steroids by the liver in portal circulation following oral administration, there was an urgent need to utilize sublingual and buccal routes to maintain the efficacy of these compounds. Furthermore, use of sublingual and buccal routes avoided frequent appointments for routine injections, thereby conserving time, minimizing discomfort, and alleviating the workload of busy practicing physicians [5,6].

In the 1950s, initial claims regarding the sublingual administration of heparins were explored to prevent degradation caused by oral gastrointestinal absorption and first-pass hepatic metabolism [7,8,9], which failed to survive critical evaluation due to the lack of reliable absorption and inconsistency of therapeutic effects, especially on blood coagulation [10,11]. Thereafter, the fact that heparins needed to cross 30–40 cellular layers to access the initial blood vessels in the lamina propria reduced the possibility of developing buccal heparin delivery systems [12].

In the early 1960s, the effects of alpha-amylase following buccal administration were examined as an anti-inflammatory agent for various conditions. However, the findings indicated that alpha amylase does not significantly reduce inflammatory responses through the buccal route due to the large size of this protein molecule and its low chance of absorption through the buccal membrane [13,14].

In the 1970s, considerable advancements were achieved in understanding the physiological and pharmacological aspects of sublingual and buccal administration systems. The work of Kates et al. improved the understanding of propranolol absorption kinetics through the sublingual route, demonstrating that this drug followed first-order kinetics with significant inter-subject variability [15]. Additionally, the study by Huston et al. revealed the localized impacts of medications on oral mucosa, which is essential for the advancement of buccal delivery systems. That research investigated the impact of different substances on the buccal mucosal potential difference (b.p.d.), emphasizing the ability of aspirin and ethanol to modify the bioelectric characteristics of the buccal mucosa [16]. Those early investigations established the foundation for the quick development and establishment of sublingual and buccal delivery systems that began in 1983.

The initial research in sublingual and buccal drug delivery systems between 1950 and 1982 faced significant biochemical and formulation-related challenges, which consequently affected the number of publications. The sublingual delivery of numerous investigated compounds, such as heparins and alpha-amylase, was hampered by poor mucosal permeability, high molecular weight, and susceptibility to degradation in the sublingual environment, ultimately limiting their therapeutic potential. Initial formulations faced significant challenges in development and patient compliance, marked by sublingual limited absorption due to limited surface area and the unpleasant taste of many medications. Early scientific understanding of mucosal membrane barriers and diffusion mechanisms, combined with suboptimal excipients, saw limited successful formulation development. The limited number of publications until the early 1980s was due to the complexities of the field, not lack of interest; this has changed with improved drug design, improved analytical tools, and better understanding of oral pharmacokinetics, consequently leading to increased research in this area [17,18].

### 1.2. Exploratory Growth Phase (1983–1993)

Starting from 1983, the number of publications concerned with the sublingual route increased notably, indicating a growing attention to embracing this approach in various investigations. To address the concerns regarding reduced systemic nitrate bioavailability resulting from hepatic enzyme-mediated degradation, innovative delivery systems were designed to bypass first-pass hepatic metabolism by glutathione-organic nitrate reductase. Among various nitrate delivery systems, sublingual and buccal nitroglycerin (BN) became crucial for the management of heart disease due to several advantages. These formulations offered a rapid reversal of side effects, in addition to BN’s main advantage of providing rapid onset of action which can be used for anginal pain, and sustained NTG therapy that remains for several hours. Another advancement in sublingual and buccal nitrate delivery systems is the elimination of the need for water during administration, which may be favored by some patients compared to oral tablets, and is well tolerated. During that time, buccal NTG was introduced to the US market as Susadrin^®^; however, that brand’s commercial failure led to its neglect by most physicians and the broader medical community [19,20,21]. Additionally, comparative evaluations of sublingual and buccal glyceryl trinitrate (SLGTN and BGTN) revealed interesting results. While Rydén and Schaffrath initially reported a statistically significant patient preference for BGTN over SLGTN in angina prevention, subsequent studies demonstrated equal efficacy for both formulations in terminating acute anginal episodes [22,23].

Furthermore, the development of sublingual and buccal delivery systems for calcium channel blockers such as nifedipine and verapamil significantly advanced non-invasive hypertension management. The effectiveness of nifedipine in the treatment of chronic essential hypertension and hypertensive emergencies has gained attention. Research conducted during that period indicated that sublingual and buccal administration of nifedipine promptly, consistently, and predictably reduced systemic arterial pressure with minimal side effects. The rapid onset and favorable safety profile of this agent presented a significant advantage over parenteral therapies, especially in urgent care settings lacking readily available continuous hemodynamic monitoring. These non-parenteral routes offered clinicians a versatile and practical approach, demonstrating high efficacy (approximately 98%) in reaching target blood pressure levels of nifedipine [24,25,26].

Due to extensive first-pass hepatic metabolism, oral verapamil initially encountered challenges due to low (10–20%) systemic bioavailability. Sublingual and buccal administration provided a pharmacokinetic advantage by bypassing this effect. Notably, sublingual and buccal verapamil exhibited a disposition profile similar to that of intravenous verapamil. This alternative delivery method utilized the extensive vascular network of the oral mucosa for more efficient systemic absorption. Early studies showed that sublingual verapamil administration resulted in substantially enhanced efficacy per milligram, yet full systemic absorption was not attained. Consequently, the buccal route represented a significant advancement in drug delivery, leveraging unique vascular and mechanical attributes to enhance absorption and closely illustrating the pharmacodynamic effects of intravenous administration. These preliminary findings from sublingual and buccal nifedipine and verapamil studies suggested that expanding the use of mucosal drug delivery systems could significantly improve clinical outcomes and patient care [27,28,29].

In the management of hypertensive crises, sublingual captopril, an angiotensin-converting enzyme (ACE) inhibitor, has emerged as a superior alternative to oral administration. The peak therapeutic effect of oral captopril is delayed to one or two hours because of its absorption from the gastrointestinal tract; in contrast, sublingual administration has overcome this delayed onset of action by omitting gastrointestinal absorption and hepatic first-pass metabolism. While pharmacokinetic profiles were similar for both routes, sublingual administration offered a more rapid time to peak concentration and faster blood pressure reduction, thus proving advantageous in acute care [30,31].

Growing interest in alternative drug delivery, especially for long-term pain management, was reflected in the increasing number of publications on sublingual and buccal methods during the 1980s. Research on this topic was limited in the 1960s and 70s; however, the 1980s and early 1990s brought a shift in perspective as healthcare professionals started acknowledging the drawbacks of conventional injectable opioid medications, particularly for patients with terminal cancer. Despite their effectiveness, injectable routes were challenged by side effects, resource demands, and impracticality for rural or home-based care. This sparked interest in sublingual and buccal administration, also advantages for bypassing first-pass hepatic metabolism and enabling fast systemic absorption via the oral mucosa’s rich vasculature. Furthermore, while these alternative routes may be advantageous for patients who cannot receive oral medications owing to bowel obstruction, vomiting, or difficulty swallowing, they are inappropriate for patients with cognitive impairment or in comatose states [32,33,34].

Morphine, an early opioid which was studied for buccal and sublingual delivery, was a primary focus of research. Bell’s findings revealed that buccal morphine demonstrated improved bioavailability over intramuscular morphine; this was evident in its higher area under the curve and slower decrease in plasma concentrations [35]. Conversely, Fisher et al.’s study yielded lower and less consistent serum levels following buccal administration, failing to reproduce the original results [36]. Pannuti subsequently determined that sublingual morphine resulted in faster and greater plasma concentrations than for oral and rectal routes, with no notable difference in the severity of adverse effects [37]. The need for better opioids and more refined formulations was highlighted due to these inconsistent results.

Consequently, the improved pharmacokinetic profiles of more lipophilic opioids like fentanyl and buprenorphine started attracting attention. Given fentanyl’s high lipid solubility and rapid, efficient oral mucosal absorption, and buprenorphine’s properties as a partial μ-opioid receptor agonist with high receptor affinity and a prolonged duration of action, sublingual delivery appears promising. Although sublingual and buccal narcotics showed promising pharmacokinetic data, their clinical use was limited and debated because their absorption and bioavailability are considerably controversial. It is noteworthy that, under controlled conditions such as alkaline pH levels, methadone, fentanyl, and buprenorphine exhibited relatively high rates of sublingual absorption. These initial discoveries paved the way for today’s dependable and clinically effective cancer pain medications [34,38].

Interest in non-invasive peptide drug delivery systems began to develop during the 1980s. By the early 1990s, the more than 150 recombinant protein drugs in clinical trials prompted researchers to explore mucosal delivery as a non-injection alternative [39]. The routes offered a potential solution to bypass the enzymatic environment of the gastrointestinal tract and avoid first-pass hepatic metabolism. Early research, however, demonstrated significant limitations in the oral mucosal delivery of peptides. In vivo permeability studies employing rabbit and dog buccal tissues demonstrated that the permeability coefficients of peptides, including thyrotropin-releasing hormone (TRH) and desmopressin (DGAVP), were significantly lower than those of small lipophilic molecules. Given the peptides’ large molecular size and low lipid solubility, which restrict their ability to pass through the lipophilic mucosal barrier, the results were not unexpected [40].

In addition to poor permeability, enzymatic degradation caused a significant challenge. Results revealed significant esterase and peptidase activity within the oral mucosa, capable of peptide degradation prior to systemic absorption. The combination of enzymatic barriers and the physicochemical properties of peptides resulted in low bioavailability compared to the parenteral route [41,42,43]. While the initial data were discouraging, they formed the foundation for future innovations, such as enzyme inhibitors, permeation enhancers, and advanced drug delivery systems such as mucoadhesive films and nanoparticles [40,44].

Considering that numerous hormones are peptides, the difficulties encountered in early peptide delivery studies translated to the development of transmucosal hormone delivery methods. These initial systems established a foundation for the development of more advanced delivery platforms by emphasizing the significance of formulation design, such as pH adjustment, cosolvent utilization, and the integration of penetration enhancers to optimize hormone permeability. These delivery methods were especially suitable for hormones, particularly those exhibiting short half-lives or poor gastrointestinal stability. Buccal delivery, despite exhibiting a slower onset than sublingual absorption, facilitated a more controlled and sustained release, thereby proving suitable for hormones demanding stable plasma levels. The relative immobility of the buccal mucosa enabled the design and implementation of mucoadhesive drug delivery systems, characterized by extended adhesion and controlled drug delivery over several hours [40]. A significant example from this period was the buccal administration of methyltestosterone with the commercial name of Metandren (Ciba) and Oreton Methyl (Schering), which validated the absorption of hormones via the oral mucosa using standard tablet formulations. These early systems laid the groundwork for more sophisticated delivery platforms by highlighting the importance of formulation design, including pH modulation, use of cosolvents, and incorporation of penetration enhancers to improve hormone permeability [40].

Even with advancements, the creation of buccal hormone delivery systems encountered various challenges. While vasopressin analogs (e.g., DDAVP) showed promise for buccal administration, their short half-lives required frequent or continuous delivery. Reaching therapeutic levels required not just permeability but also prolonged retention of the dosage form at the absorption site—a process often complicated by salivary flow, eating, and speaking. Besides, for hormones like insulin that have very short half-lives, researchers found that buccal delivery was only feasible with continuous dosing and penetration enhancers in order to reach its therapeutic level [45]. Despite this, adhesive patches [46,47,48,49] and controlled-release tablets [50,51] emerged as promising technologies during this period, delivering hormones for 6–12 h comfortably and ensuring patient compliance. These advancements in hormone therapy also set the standard for future transmucosal delivery system design.

### 1.3. Diversification and Discovery Phase (1994–2009)

The period between 1994 and 2009 witnessed variable research efforts in sublingual and buccal drug administration. The annual publication rate fluctuated, ranging from a minimum of five to a maximum of 12 studies, with an annual average of approximately six. While research activity fluctuated, this period produced considerable early contributions to numerous areas. The use of these delivery routes was explored for a range of therapeutic agents, such as buprenorphine, fentanyl, and oxycodone (opioid use disorder and analgesia), estrogens and testosterone (in hormone replacement therapies), and misoprostol and oxytocin (labor-inducing drugs). Additional investigations also included oral administration of peptides, for example insulin, and the exploration of oral immunotherapy. Simultaneously, scientists investigated the mechanisms of taste and methods to improve the taste of medications, crucial for patient well-being and adherence. Advancements were also made to comprehend the buccal and sublingual absorption and metabolism of pharmaceuticals. In the next sections, we’ll delve deeper into these developments, illustrating them with key studies from this pivotal period.

Drug transport across the sublingual and buccal tissues is influenced by better understanding of the anatomical and physicochemical absorption parameters. Sublingual absorption is primarily influenced by drug passive diffusion across the lipid membrane, by drug lipid solubility, by drug molecular weight, drug ionization, and the surrounding pH. Active transport is generally absent in the oral mucosa, thereby limiting the absorption of substances requiring carrier-mediated mechanisms. Osmosis plays a central role in drug movement, with small, water-soluble molecules diffusing freely between tissues [52,53]. Salivary gland acid stimulation improves absorptive capacity through vasodilation and increased fluid secretion. Sublingual drug delivery is optimal for compounds exhibiting moderate molecular weight, balanced aqueous and lipid solubility, partial non-ionization at physiological salivary pH (approximately 6.0), and favorable water/oil partition coefficients (ranging from 40 to 2000). In general, this route is especially beneficial for drugs with extensive first-pass metabolism, such as nitroglycerin, fentanyl citrate, and estradiol [53,54,55].

Increased interest in sublingual and buccal drug administration during the 1990s and early 21st century led researchers to investigate the subsequent behavior of such drugs within the oral mucosa. This period represented a significant advancement in our comprehension of the pharmacokinetic profiles of these delivery methods, encompassing not only the rate of drug absorption but also the consistency of systemic drug delivery and comparative therapeutic efficacy relative to established approaches. Prior to a comprehensive understanding of their pharmacokinetic profiles, several drugs, such as captopril and phenazocine, exhibited promising clinical outcomes. Sublingual administration of other agents, including nifedipine and specific β2-agonists, was initially considered viable; however, subsequent research demonstrated inadequate absorption and efficacy, resulting in their diminished clinical application. Despite these obstacles, the field showed consistent progress. Research into new formulations, including buccal nitroglycerin, advanced the field by proving the possibility of simultaneous rapid onset and sustained release using the mucosal route [56].

The evolution of sublingual and buccal drug delivery progressed from initial experimentation to a targeted pursuit of practical solutions during the 1990s. The research expanded beyond basic testing of oral mucosa drug absorption to encompass the design of patient-friendly and consistently effective drug delivery systems. This shift led to a significant increase in creative output in the field of dosage form development. A range of delivery systems, including tablets, lozenges, sprays, chewing gum, and medicated lollipops, was investigated. However, a significant drawback of these early formulations was the leakage of drugs into the gastrointestinal tract, thereby diminishing their efficacy and compromising the objective of targeted mucosal delivery [57].

To address this challenge, researchers investigated mucoadhesive technologies; specifically, formulations engineered for controlled drug release via mucosal adhesion. Hydroxypropyl cellulose and Carbopol proved to be indispensable components, providing both adhesive properties and enhanced comfort. Such advancements facilitated the creation of more complex systems, including the 3M Cydot^®^, a flexible, biocompatible gum disk, and TheraTech’s OTS^®^ bilayer tablets, which enabled direct peptide (e.g., GLP-1) delivery into the bloodstream. An increasing understanding of patient needs, especially among those intolerant to oral or parenteral administration, was reflected in these systems. Emphasis was placed on a patient-centered design, prioritizing comfort, discretion, and ease of use [47,58,59,60]. Despite advancements in formulation science, a significant biological obstacle remained: the buccal mucosa. The thin, moist, and highly vascularized tissue, although readily accessible, significantly blocked drug absorption. Researchers initiated investigations into drug transport across this barrier via in vitro diffusion studies and in vivo experimentation.

A range of in vitro model systems simulating the human buccal environment have been employed by researchers to aid in studies of mucosal drug transport. Porcine buccal mucosa, while structurally similar to human tissue, presented challenges due to its comparatively high permeation and the fragility inherent in its preparation, leading to variability and limitations. Porcine esophageal mucosa, among other alternative tissues, demonstrated superior permeability, ease of handling, and comparable epithelial lipid composition, thus proving to be valuable substitutes. Comparative analysis employing permeation markers, namely arecoline and estradiol, demonstrated superior buccal permeability correlation between human mucosa and porcine oral base mucosa compared to porcine cheek mucosa [61,62,63,64]. Human vaginal mucosa, which shared similar physiological and lipid properties with buccal tissue, has also proven effective in penetration studies [65]. In addition to chicken and golden hamster cheek pouches, cultured cell lines—like TR146 from human buccal carcinoma cells—are used in experiments, thanks to advances in tissue engineering. TR146 demonstrated permeability, enzymatic activity, and responsiveness to environmental stimuli closely mirroring that of human buccal mucosa, thereby establishing it as a dependable and reproducible platform for in vitro mucosal penetration studies [66,67,68,69,70].

The use of animal models (rabbits, rats, dogs, and pigs) was fundamental to early pharmacokinetic investigations, but such models were not without limitations. Ensuring consistent contact between the dosage form and the mucosa proved challenging, necessitating sedation or physical restraint to achieve accurate dosing. Bioavailability studies using peptides, like leuprolide, can be misleading in dogs due to their substantially higher oral mucosal permeability than humans. Monkeys also have a more similar mucosal structure, but their cost and handling make them impractical for formulation testing [71]. Furthermore, investigations in rabbits concerning insulin demonstrated bioavailability variability (3–5%) contingent upon pH, whereas thyrotropin-releasing hormone (TRH) exhibited a bioavailability of only 2%. Buccal administration of heparin in canines achieved therapeutic concentrations; however, insulin failed to obtain a hypoglycemic effect. Pigs exhibited just 2% absorption of FITC-dextran, which rose to 13% when bile salt enhancers were introduced. This highlights the barrier function of the mucosa, and the difficulties associated with ensuring consistent drug contact in animal models [72,73,74,75].

Conversely, initial human trials began to yield more reliable outcomes. For instance, TRH resulted in significant increases in the levels of thyrotropin and prolactin in the blood [48]. The buccal administration of buprenorphine resulted in a bioavailability of 28%, which was lower than that of sublingual delivery but remained clinically significant, with serum concentrations maintained for as long as 16 h [76,77]. Testosterone administered buccally caused a 3.3-fold increase compared to baseline levels, while atipamezole given as a buccal spray achieved 33% bioavailability—significantly better than its oral formulation, although some local irritation was observed [78]. Other medications, like clonazepam [79] and fentanyl [80], showed similar or better outcomes compared to nasal or oral administration, with fentanyl lollipops offering effective pain management for pediatric patients. Morphine sulfate demonstrated a 30% absorption rate from buccoadhesive tablets, whereas prosidol provided superior pain relief compared to its oral version [81]. Even medications such as GLP-1, a peptide that is usually difficult to administer, reached a bioavailability of 6–7% in human volunteers [59,60]. The findings not only confirmed the promise of buccal and sublingual methods but also emphasized the significance of formulation design and user-focused testing.

To enhance absorption, researchers during the 1990s investigated various strategies for improvement. They examined chemical penetration enhancers—including bile salts, fatty acids, surfactants, and chelators—to determine their effectiveness in temporarily relaxing the tight junctions between epithelial cells or boosting membrane fluidity. Certain substances, such as sodium deoxycholate, demonstrated notable potential in enhancing the uptake of large molecules. Various physical techniques were developed, such as iontophoresis, which employed a gentle electrical current to help transport medications through the mucous membranes [82]. Scientists also conducted experiments with prodrugs and enzyme blockers to protect delicate molecules from breaking down in the mouth. A significant instance from this period is nitroglycerin, commonly utilized in the management of angina pectoris. While not always specifically classified as a prodrug, nitroglycerin acts as one: it is processed in the body to produce nitric oxide, the active ingredient that causes vasodilation and alleviates symptoms. Its low molecular weight and high lipophilicity made it suitable for delivery sublingually, and it became one of the first and most successful medications given through this method. By the 1990s, numerous products containing nitroglycerin—such as Suscard, Nitrobid, and Nitromex—were available, providing quick relief via sublingual absorption [72].

Additional prodrugs also gained popularity during this decade. Isosorbide mononitrate, a different nitrate utilized for treating angina, was found in products such as Imdur, Isordil, and ISMO, depending on metabolic conversion to its active form following mucosal absorption. In a similar way, methyl testosterone, which is utilized in hormone replacement treatments for issues such as hypogonadism and delayed puberty, was available in sublingual forms like Oreton Methyl, Testred, and Virilon. Besides, sublingual buprenorphine provided a non-injection option for pain relief, while nicotine, ergotamine, and lorazepam were used sublingually for smoking cessation, migraine relief, and anxiety or insomnia treatment, respectively [72]. These examples demonstrate how the 1990s represented a pivotal moment—not just in the creation of new delivery systems but also in the strategic application of prodrug chemistry to improve the efficacy of buccal and sublingual drug delivery (Table 1).

By the conclusion of the decade, the area had achieved notable progress. Buccal and sublingual drug delivery systems were transitioning from the laboratory to clinical application. Pharmaceuticals such as Nitrolingual^®^ spray and fentanyl lollypops have shown how these methods of delivery can provide quick relief in emergencies or palliative care scenarios [83]. At the same time, the creation of mucoadhesive patches and bilayer tablets facilitated prolonged drug release, enhancing convenience and adherence. These advancements were particularly beneficial for groups with difficulties in swallowing, including older adults and patients recovering from surgery [72]. As an increasing number of products became available and regulatory structures started to develop, the idea of sublingual and buccal administration as common therapeutic alternatives transitioned from a distant aspiration to an achievable reality.

By the early 2000s, the field’s development revealed a significant disparity between the scale of research and the translation of that research into commercial products. Despite more than 90 companies’ engagement in developing oral cavity drug delivery systems, such as buccal and sublingual routes, fewer than 50 products reached the US market. A substantial portion of these were essentially modifications of the same active pharmaceutical ingredients (APIs), such as various flavors or doses of nicotine chewing gum. The disparity emphasized a continuing problem: while patents and innovative formulations increased significantly, relatively few transmucosal medications secured regulatory approval or broad clinical application. Although patents documented exploration of thin films and bite capsules, their commercialization remained limited [84,85].

The 1996 FDA approval of fentanyl Oralet™, the first formulation designed for oral transmucosal opioid delivery in children, provided a painless, child-friendly alternative to injections and paved the way for subsequent advancements [86]. This was followed by the commercial success of Actiq^®^ (fentanyl transmucosal lozenge) in 1998, which confirmed the clinical feasibility of transmucosal administration for powerful, rapidly acting medication. Physicochemical strategies, such as in situ conversion to enhance lipophilicity, were implemented in these formulations to improve mucosal permeability and enable rapid systemic effects. Yet, obstacles remained, primarily within pediatric applications requiring careful consideration of taste, precise dosing, and patient eagerness. Although regulatory and clinical challenges remained, the market for buccal and sublingual drug delivery systems was becoming more diverse by the mid-2000s. Cephalon’s Fentora^®^, a buccal effervescent tablet launched in 2006, represented a move towards more advanced drug formulations with faster systemic absorption. In the treatment of breakthrough cancer pain, Fentora demonstrated improved bioavailability over its precursor Actiq^®^, through the in-situ transformation of fentanyl citrate into a more lipophilic free base [87,88].

The approval of buprenorphine-based treatments for opioid dependence, namely Subutex^®^ and Suboxone^®^, occurred concurrently. Suboxone^®^, incorporating naloxone, was designed to deter injection misuse. These approvals began a significant turning point, enabling transmucosal systems to tackle not only acute pain, but also chronic conditions and public health challenges [85]. Significant advancements included the utilization of Buccastem^®^ (prochlorperazine) for nausea, Striant^®^ for testosterone replacement therapy, and Loramyc^®^ (miconazole) for oropharyngeal candidiasis. Each product employed mucoadhesive or extended-release technologies to enhance therapeutic efficacy. Meanwhile, the approval of Sativex^®^, a buccal cannabis spray, in Canada for the treatment of cancer pain and multiple sclerosis signifies an increasing acceptance of novel active compounds delivered via the buccal route. The use of unlicensed formulations, including buccal midazolam (Epistatus^®^), has shown promise in managing seizures in children, offering a minimally invasive alternative to rectal diazepam administration [85,89].

Advancements in that time were countered by regulatory obstacles—most notably, the FDA’s rejection of Emezine^®^ due to pharmacokinetic concerns—thereby emphasizing the difficulties in achieving a balance between efficacy, safety, and the variability observed in patients [85]. Toward the end of the 2000s, the development pipeline for buccal and sublingual drug delivery systems reached a peak of activity, featuring numerous products in the later phases of development and several having already attained regulatory approvals. The development of NovaDel’s ZolpiMist™ (zolpidem oral spray) and Nitromist™ (nitroglycerin lingual spray) highlighted the increasing focus on reformulating familiar drugs to facilitate rapid transmucosal absorption. Some projects, however, did not achieve immediate success. Ondansetron buccal spray, in particular, faced difficulties arising from formulation instability, leading to subsequent reformulation and new licensing arrangements. Besides, Transcept Pharmaceuticals developed a low-dose zolpidem lozenge to address insomnia during the night, providing patients with a self-administered option exhibiting reduced side effects [85]. Generex’s oral insulin spray, approved in Ecuador in 2005, marked a pivotal advance as the first non-injectable insulin product. This was made possible by employing micellar encapsulation and buccal absorption to prevent degradation within the gastrointestinal tract. Additionally, the company investigated the buccal delivery of metformin using chewing gum as a method of mitigating gastrointestinal adverse effects [85]. These innovative findings indicated the significant therapeutic advantages of transmucosal delivery, particularly for drugs exhibiting instability within the gastrointestinal tract or requiring a prompt therapeutic response.

In the early 2000s, sublingual and buccal administration of misoprostol emerged as a promising area of research for various medical applications, including abortion, cervical ripening, and labor induction [90]. Sublingual administration, especially, exhibited significant pharmacokinetic benefits. Misoprostol tablets, characterized by high solubility, demonstrated complete dissolution sublingually within 20 min, resulting in expedited absorption. Sublingual administration indicated the highest peak plasma concentration, the most rapid time to peak (approximately 30 min), and the greatest systemic bioavailability in comparison to oral and vaginal routes with the addition of preventing direct cervical exposure, likely diminishing the risk of uterine hyperstimulation, as revealed by comparative studies. The improved performance is a consequence of the sublingual mucosa’s rich vascularization and the elimination of first-pass hepatic metabolism [91,92,93,94]. In comparison, the buccal route demonstrated a slower absorption rate, resulting in a Tmax of 75 min and an AUC roughly half that observed with vaginal administration. While the buccal administration yielded lower serum concentrations than the sublingual route, as evidenced by one study reporting a fourfold increase in sublingual area under the curve (AUC), it remained clinically efficacious and presented a viable alternative, especially in situations that sublingual delivery cannot be used [95,96,97,98]. The results emphasized the critical role of route selection in maximizing the therapeutic efficacy of misoprostol, thus necessitating additional comparative research to optimize clinical guidelines.

Moreover, multiple studies during the 1990s investigated the buccal absorption of numerous polypeptide drugs [99]. Gastrointestinal peptides (e.g., secretin, substance P), pancreatic hormones (insulin, glucagon), pituitary polypeptides (adrenocorticotropins, growth hormone), hypothalamic-releasing hormones (gonadorelin, somatostatin), and other bioactive peptides such as calcitonin and interferons were among the new buccal delivery systems developed in this period [100,101,102]. Although progress has been made, delivering peptides via the buccal mucosa is difficult because of their inherent instability, large size, water-loving nature, and susceptibility to enzymatic breakdown. The permeability and bioavailability of these compounds are limited by these factors. Although conventional methodologies, including chemical modification, prodrug formation, and the utilization of mucoadhesive and permeation-enhancing systems, remained popular for absorption enhancement, innovative technologies such as liposomal delivery systems presented themselves as superior alternatives to addressing these limitations by increasing local drug concentration and decreasing systemic drug levels [103,104].

Buccal drug delivery systems utilizing liposomal formulations offer a novel method for encapsulating both hydrophilic and hydrophobic drugs, such as insulin peptides [104,105,106,107,108]. However, investigations using diabetic rat models have demonstrated restricted systemic efficacy with liposome-encapsulated insulin (LEV-INS) delivery, indicating potential limitations in drug transport beyond superficial epithelial tissues [109]. For performance enhancement, liposomes have been integrated with mucoadhesive polymers, including polymethylmethacrylate, to improve their stability and retention within the oral mucosa. Moreover, the integration of protease inhibitors, such as aprotinin, within liposomal delivery systems, including those designed for Factor VIII, has been investigated to mitigate enzymatic degradation of peptide therapeutics [110]. The efficacy of liposomes as a buccal delivery system, while promising for localized applications, required further optimization to effectively deliver peptides systemically.

Sublingual immunotherapy (SLIT) was first implemented in European clinical practice in the absence of rigorous clinical trial data. Early evaluations of the therapy relied mostly on small, uncontrolled, or open-label studies. By 2000, only seven trials satisfied the methodological criteria for meta-analysis inclusion, indicating a deficiency in the quality of early investigation [111]. Although substantial, well-structured research was advocated, most studies remained limited in scope until a 2005 meta-analysis. This meta-analysis, containing 21 trials and 959 participants, offered initial support for SLIT’s effectiveness in mitigating allergic symptoms [112]. Subsequently, three large-scale, double-blind, placebo-controlled trials involving over 1600 patients that assessed a standardized grass-pollen tablet (Phleum pratense) were published in 2006, representing a critical moment in the field. These studies indicated a 35–40% decrease in clinical symptoms and a comparable reduction in the use of this sublingual immunotherapy during the first year of treatment. A significant proportion of patients undergoing this treatment reported improved symptoms; the safety profile was positive, with localized irritation being the most common adverse event [113,114,115]. As shown in Table 2, following these findings, the grass-pollen tablet (Grazax^®^, ALK-Abelló) received regulatory approval in Europe, representing the initial standardized sublingual immunotherapy (SLIT) product to be integrated into clinical practice [116]. While other allergen extracts, including cat dander, were examined, they lacked approval and comprehensive study [117]. Throughout this period, SLIT experienced a rise in usage within Europe, predominantly amongst patients demonstrating mono- or oligo-sensitization to seasonal allergens, while no SLIT products received approval from the US Food and Drug Administration (FDA) [116].

Table 3 shows the FDA-approved sublingual and buccal drug products in the US. The table highlights the different delivery systems, bioavailability, and therapeutic applications for currently marketed and discontinued formulations. For each active pharmaceutical ingredient, only the earliest approved example of each dosage form was selected to minimize redundancy and reflect on the historical development of transmucosal drug delivery systems [120].

### 1.4. Innovation and Integration Phase (2010–2025)

**Figure 1:**
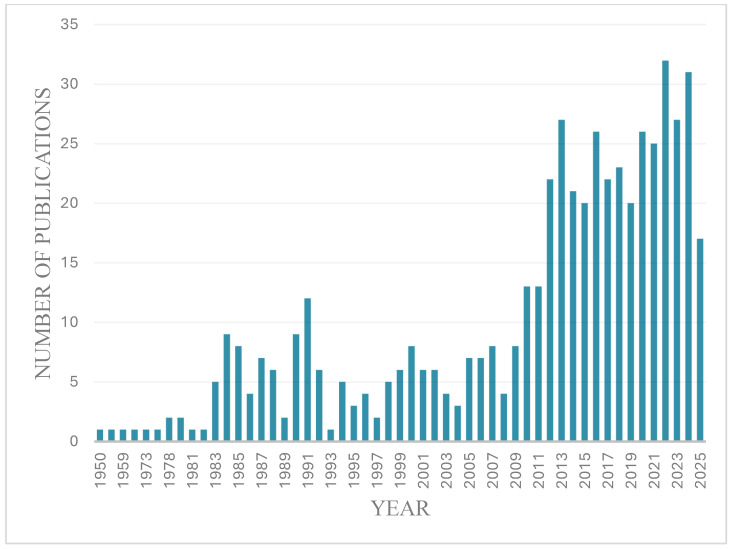
Annual number of publications indexed in PubMed from 1950 to 2025 using the search query “sublingual and buccal”.

Between 2010 and mid-2025, scholarly output on sublingual and buccal drug delivery systems exhibited considerable and consistent growth, increasing from 13 publications in 2010 to a high of 32 in 2022, with subsequent numbers of 27 and 31 for 2023 and 2024, respectively, and 17 by June 2025 (Figure 1). This phase was marked by a notable increase in the diversity of therapeutic applications and formulation techniques. Key advancements involved the development of nanoparticles, and multilayered mucoadhesive systems, in addition to the creation of pediatric formulations like rapidly dissolving tablets and films. Sublingual and buccal administration has seen increased investigation for immunotherapy, vaccine delivery, and a broad spectrum of therapeutic agents, such as steroids, antifungals, cannabinoids, antidepressants, antipsychotics, and narcotics (e.g., buprenorphine and apomorphine). Continued advancements were also observed in the application of misoprostol, novel formulations of fentanyl and diazepam for pain and seizure control, and the introduction of buccal vitamin D3 sprays. These advancements together demonstrate a growing area of research focused on enhancing mucosal drug delivery to achieve local and systemic therapeutic benefits (Table 4).

Sublingual and buccal drug delivery transitioned from experimental to commercial viability in the early 2010s. This period witnessed the development of more accessible formulations, especially for pediatric patients and marked by a substantial rise in both research endeavors and product approvals. A notable advancement was the European approval of Breakyl (fentanyl buccal film), utilizing BEMA^®^ (BioErodible MucoAdhesive) technology. This bioerodible film, characterized by its thin profile, exhibits strong adhesion to the buccal mucosa and ensures expeditious fentanyl delivery in the treatment of breakthrough cancer pain. Onsolis, its US and Canadian equivalent, employed the same technology. Subsequently, BEMA Buprenorphine successfully concluded Phase III clinical trials for chronic pain management, thereby providing a non-invasive alternative to conventional opioid treatments. Subsys, a sublingual fentanyl spray providing rapid onset and ease of use for breakthrough cancer pain, represented a major advancement [121].

As the first transmucosal immediate-release fentanyl product in the US to be approved under a Risk Evaluation and Mitigation Strategy (REMS), the rapidly dissolving sublingual fentanyl tablet Abstral also gained market access in Europe. Under the EU’s Pediatric Use Marketing Authorization (PUMA), Buccolam (midazolam oromucosal solution) was approved for the treatment of acute, prolonged convulsive seizures in Pediatrics. A new buccal insulin spray, delivered by the RapidMist™ device, has been introduced by Generex Oral-lyn as a treatment for metabolic disorders. Recombinant human insulin was directly delivered to the buccal mucosa via this formulation, preventing pulmonary absorption. Clinical trial data indicated a significant HbA1c reduction in the absence of adverse effects. In several European countries, the oromucosal spray Sativex, which contains THC and CBD, received approval for managing multiple sclerosis-related spasticity; it has also progressed to Phase III clinical trials in the US for the treatment of cancer pain. Moreover, the approval of Suboxone buccal tablets, a buprenorphine and naloxone combination, for opioid dependence was followed by approval of a sublingual film formulation utilizing MonoSol Rx’s PharmFilm^®^ technology. Intermezzo, a sublingual zolpidem tartrate tablet, has been also approved as a treatment for insomnia [121].

In the early 2010s, researchers increasingly explored chemical and physical methods to overcome the buccal mucosa’s natural barriers. To improve mucosal permeability, studies investigated chemical absorption enhancers including DDAIP HCl, Br-iminosulfurane, and azone, with DDAIP HCl demonstrating notable efficacy for lidocaine and nicotine delivery [122]. Meanwhile, physical techniques like iontophoresis and electroporation explored as methods for electrically delivering drugs across the mucosa [123]. These innovations led to the creation of advanced systems, notably the IntelliDrug device: a miniaturized intraoral appliance providing controlled and continuous delivery of medications such as naltrexone and galantamine. This highlights the potential of integrated electronic platforms for buccal-route chronic disease management [124,125].

A significant increase in the scientific literature during the early 2010s indicates a considerable interest in nanoparticle drug delivery systems for buccal and sublingual routes of administration. The potential of nanoparticles to improve oral drug delivery by enhancing solubility, protecting drugs, and controlling release was explored by researchers, targeting the challenges posed by the oral mucosa [126,127]. The number of publications focusing on lipid and polymer-based nanoparticles for oral mucosal delivery increased notably between 2010 and 2015. Studies most frequently investigated lipid and polymer nanoparticles, demonstrating that surface modifications like PEG coatings enhance mucus penetration, decrease mucus interaction, and improve lymph node distribution [128,129]. Various dosage forms—gels, sprays, tablets, films, and patches—were created using these systems to enhance mucosal contact and drug absorption. Studies using 200 nm FluoSpheres^®^ polystyrene nanoparticles, for instance, showed penetration of the buccal epithelium and underlying connective tissue in human and porcine ex vivo models [130].

Teubl et al. (2013) similarly reported that 200 nm neutral polystyrene nanoparticles exhibited greater penetration depth in porcine buccal mucosa than smaller particles, indicating size-dependent permeability [131]. The impact of surface charge was also examined as a critical role: 200 nm cationic nanoparticles showed deeper buccal tissue penetration than anionic counterparts, according to Roblegg et al. (2012); however, other studies proposed that anionic particles diffuse through mucus more easily because of weaker electrostatic interactions [132]. Masek et al. (2017) innovated a nanofiber-based mucoadhesive film with embedded nanoparticles, showing sustained drug release and mucosal penetration in vivo pig studies [129]. The importance of nanoparticle composition, size, charge, and formulation base for delivery efficiency was highlighted by these findings. In the field of nanoparticulate systems, solely ropivacaine liposomal gel has progressed to clinical trials (Phase I) for sublingual/buccal topical anesthetic administration [133].

These advances in nanocarrier drug delivery systems have significantly expanded on the options for buccal and sublingual routes, especially in cases of molecules with limited permeability or lipid solubility. Delivery platforms such as tablets, wafers, and films which incorporate nanoparticles exhibited extended mucosal residence time, resistance to salivary wash-off, and improved particle stability. Studies indicated that solid lipid nanoparticles (SLNs) and PEGylated liposomes offer potential for the modulation of release kinetics, enhanced permeation, and targeted delivery to immunological cells within the sublingual epithelium. Polymeric mucoadhesive systems, including Eudragit, HPMC, and Carbopol, offer tunable hydration, adhesion, and drug release properties, as demonstrated through formulations containing acyclovir, lornoxicam, and curcumin. However, these novel technologies have inherited limitations linked to their complex preparation, size-dependent release, tissue distribution variability, and unexpected excipient performance. Overall, these systems altogether create a flexible platform to overcome the physiological challenges of transmucosal drug delivery; however, further improvements are necessary to expand their use in delivering various macromolecular therapeutics [118,129,134,135,136,137].

A marked increase in the number of sublingual and buccal drug formulations undergoing clinical trials and market introduction has been observed in recent years, indicating increased interest in these non-invasive administration methods for acute and chronic disease management. A wide range of therapeutic areas have been addressed by the approved formulations, including sedation (e.g., sublingual lorazepam), insomnia (melatonin), angina (isosorbide dinitrate), epilepsy (midazolam), and vitamin B12 deficiency (formulated as tablets, sprays, films, and lozenges). Additional significant examples to consider include zolmitriptan, indicated for the treatment of cluster headaches, and riluzole, utilized in the management of amyotrophic lateral sclerosis. Furthermore, there are investigational pharmacological agents, such as ALKS 5461, which are being explored for major depressive disorders, along with UISH001 for urinary incontinence [138]. The various dosage forms—solid (e.g., tablets, films, and lozenges), liquid (e.g., sprays and oral drops), and semi-solid (e.g., gels)—are formulated to rapidly dissolve or disintegrate within the oral cavity, thus enabling rapid mucosal absorption independent of water. The majority of innovations have centered on improving traditional formulations through the use of mucoadhesive polymers or permeation enhancers to optimize mucosal retention and drug absorption [139]. Synthetic polymers such as cellulose derivatives and poly(acrylic acid), and natural agents including chitosan, hyaluronic acid, and agarose are among these [140]. Furthermore, while slow-dissolving, multilayered films offer sustained release, mucosal irritation, dislodgement during consumption, and unwanted gastrointestinal adhesion remain a concern [133].

The period between 2010 and 2025 saw the rise of sublingual and buccal vaccination as promising alternatives to nasal and injectable methods, primarily due to safety issues with nasal vaccines (like Flumist^®^) and the increasing preference for painless delivery [141,142]. Sublingual administration, a known method of allergy immunotherapy, has been attracting interest for its capacity to stimulate both systemic and mucosal immunity in distant sites like the respiratory, gastrointestinal, and reproductive systems [143,144]. The oral mucosa’s tolerogenic nature hindered vaccine development, requiring potent adjuvants and innovative delivery to overcome immune suppression and boost antigen presentation [145].

Progress in antigen formulation, delivery technologies, and mucosal immunology drove the rise in publications during this period. Studies investigated various viral delivery methods, including viral vectors (e.g., adenovirus, baculovirus), virosomes, and virus-like particles (VLPs), often boosted by adjuvants like α-GalCer, CpG-ODN, c-di-AMP, and cholera toxin B subunit (CTB) to improve the immune response [146]. For example, co-administering freeze-dried VLP-based vaccines against Group A streptococcus with CT enhanced salivary SIgA responses. Recombinant protein vaccines, although safer, needed structural changes such as PEGylation or PASylation to better cross mucous membranes and be absorbed by APCs [147,148]. Needle-free injectors (MucoJet, Syrijet, microneedle arrays) were developed in this period to improve antigen delivery via mucosa, boosting retention and minimizing saliva washout [149]. A clinical trial (NCT03629041) demonstrated that microneedle patches are safe and may be useful in delivering vaccines [150]. The sublingual delivery of vaccines has recently been extended to include solid forms of HIV-1 antigens, specifically gp41 and gp140, through the application of thermostable virosomes and mucoadhesive platforms. By combining antigens and adjuvants in one particle, these formulations decreased the reliance on cold chain logistics; yet, maintaining antigen stability at room temperature remained a challenge [151].

Simultaneously, mucoadhesive drug delivery systems, including films, tablets, and gels, were designed to extend antigen exposure to the sublingual mucosa and reduce dilution. Using these solid and semi-solid scaffolds improved the thermostability and preserved the bioactivity of sensitive proteins, viruses, and enzymes. Chitosan, cyclodextrins, and stabilizing polymers increased mucoadhesion and permeability, leading to tailored drug release and enhanced immune responses [152,153,154]. Another illustrative example is a free-standing membrane fabricated via Layer-by-Layer assembly of chitosan and hyaluronic acid, subsequently modified with bioactive proteins such as ovalbumin. The scaffold demonstrated superior mucosal retention and epithelial penetration in animal studies, thereby offering a viable alternative to liquid protein formulations, which frequently exhibited limited mucosal contact and unreliable dosing [155]. Furthermore, the successful penetration of the keratinized mucosa by mucoadhesive Layer-by-Layer patches containing poly(lactic acid) nanoparticles loaded with viral proteins and adjuvants resulted in immune responses comparable to gold-standard mucosal vaccines, which signified a notable progress in needle-free immunization [156]. Optimizing the fabrication process was crucial to prevent antigen denaturation and immunogenicity loss, as shown in studies by Bajrovic et al. [157] and Amacker et al. [158]. These results showed that the integrity of the vaccine formulation is key to its effectiveness [152].

While buccal and sublingual drug delivery offers advantages like bypassing hepatic first-pass metabolism and enabling rapid absorption, significant physiological and physicochemical challenges remain, particularly for macromolecular drugs. A major barrier to buccal drug delivery is the stratified squamous epithelium (500–800 μm), particularly its upper-level cells, which are lacking tight junctions and are rich in lipid-containing granules which restrict passage of large hydrophilic molecules. While many studies have explored the role of permeation enhancers (e.g., surfactants, bile salts, and fatty acids), significant obstacles continue to hinder progress. Furthermore, although P-glycoprotein expression is less pronounced in buccal tissue than in intestinal mucosa, the presence of CYP3A4 at comparable levels may influence buccal bioavailability [159,160,161,162,163,164,165]. Sublingual delivery, due to the reduced thickness of the epithelial tissue (100–200 μm), allows for more direct access to systemic circulation and greater control of the drug’s microenvironment. However, challenges remain for macromolecule delivery due to pH fluctuations, limited surface area, and enzyme exposure. Optimization of physicochemical properties (such as pKa, solubility, and logP) and local pH may enhance drug absorption, as demonstrated in studies using fentanyl effervescent formulations [1,53,165,166].

Advanced imaging technologies finally helped us significantly understand how sublingual vaccines are delivered. The utilization of whole-body fluorescence imaging, confocal microscopy, and tissue clearing methods allowed for the real-time monitoring of antigen trafficking, lymphatic drainage, and germinal center development in the draining lymph nodes [148]. These insights not only shaped the formulation but also highlighted the need to combine delivery innovation and immunological precision. Advancement in imaging techniques enhanced sublingual and buccal vaccine research and notable publications were due to these advances.

## 2. Future Directions

Recent progress in drug efficacy and patient-better outcomes was driven by the integration of mucoadhesive technologies, innovative polymers, permeation enhancers, and personalized approaches. Material science, biotechnology, and computational design will shape the future of sublingual and buccal drug delivery. The expanding interest in noninvasive therapeutics is leading to several advancements that will strengthen their potential. mRNA-based treatments, known for their role in the COVID-19 pandemic, may be adaptable for mucosal delivery. Significant progress in stability, encapsulation, and mucosal transport is needed, but this approach could provide a self-administered alternative to injections, particularly in low-resource areas. Scientists are also actively exploring gene delivery platforms. The buccal and sublingual mucosa’s direct access to systemic circulation makes them appealing for therapies needing rapid delivery. Microneedle arrays, dissolvable films, and nanoparticle carriers are under investigation for their potential to enhance mucosal delivery and protect sensitive genetic material. Moreover, buccal administration could become more important in immunotherapy and vaccine development. Due to its high concentration of Langerhans and dendritic cells, the oral mucosa is an excellent location to stimulate local and systemic immunity. Needle-free vaccination strategies, safer, simpler, and potentially more appealing to patients, are now within reach [167,168,169,170].

The role of artificial intelligence in pharmaceutical development is rapidly expanding. Despite their limited current use in buccal and sublingual drug delivery, AI algorithms have already shown potential for optimizing formulations, predicting bioavailability, and screening materials. The development of patient-specific treatment formulations may be enabled by future AI-integrated systems utilizing real-time data streams from wearable biosensors and digital health platforms. Such technologies may facilitate the development of adaptive therapies responsive to physiological changes, environmental factors, or individual genetic makeup, thereby advancing personalized medicine in mucosal drug delivery [171].

Successful market entry for these technologies necessitates regulatory advancements and interdisciplinary collaboration, in addition to effective formulations. Optimized approval processes, comprehensive patient education, and robust compliance measures will facilitate equitable access to advanced therapies for patients. Sublingual and buccal drug delivery could become central to future therapeutics if we invest in translational research and smart design [172,173,174,175,176].

## 3. Conclusions

This review explored the historical, scientific pathway and evolution of sublingual and buccal drug delivery, stressing their important clinical relevance and adaptability. Unlike oral administration, which often leads to slower effects and less drug absorption because of digestion and liver metabolism, buccal and sublingual delivery methods avoid liver metabolism, resulting in faster absorption into the bloodstream. The high vascularity and thin mucosa under the tongue allow for rapid absorption with sublingual delivery, as seen with nitroglycerin and captopril, which reach peak plasma concentration within minutes. Buccal drug delivery, although slower, offers extended release, making it appropriate for drugs like buprenorphine and morphine. The main advantages of these delivery routes—rapid onset, improved bioavailability, and better patient compliance—are consistently seen, from their traditional medicinal roots to their current use in systemic and localized therapies. Specifically, buccal systems offer promising peptide delivery and immunotherapy, and sublingual systems provide viable alternatives for allergen and vaccine administration. In conclusion, exploring and understanding the history of sublingual and buccal drug delivery systems enabled researchers to explore this route, select the most suitable animal models, develop the pharmacokinetic parameters, and identify novel approaches to overcome the absorption limitations associated with the route, and brought this area of research into the current maturity level.

## Figures and Tables

**Table 1:** Prodrugs utilized in buccal and sublingual drug delivery.

Prodrug	Benefit	Mechanism
Nitroglycerin	Enhanced sublingual membrane permeability	Enhanced physical chemical properties such as low molecular weight and high lipophilicity improved permeability across sublingual membrane
Isosorbide mononitrate	Metabolite activation post-absorption	Designed to remain stable during transit and become activated after mucosal uptake
Methyl testosterone	Protection from enzymatic degradation	Enhanced stability in the oral cavity before reaching systemic circulation

**Table 2:** Approved buccal and sublingual drug products commercialized by pharmaceutical companies in Europe [118,119].

Drug Name	Brand Name	Delivery Type	Company	Indication	Therapeutic Class	Status
House dust mites allergen extract	Acarizax^®^	Fast-dissolving tablet (Sublingual, Lyophilized)	ALK-Abelló (Horsholm, Denmark)	Allergic rhinitis; Asthma	Allergen immunotherapy	RX
	Actair^®^	Tablet (Sublingual)	Stallergenes Greer (Antony, France)			
Grass pollen allergen extract	Grazax^®^	Fast-dissolving tablet (Sublingual, Lyophilized)	ALK-Abelló (Horsholm, Denmark)	Seasonal allergy	Allergen immunotherapy	RX
Betula verrucosa allergen extract	Itulazax^®^	Fast-dissolving tablet (Sublingual, Lyophilized)	ALK-Abelló (Horsholm, Denmark)	Allergic rhinitis; Conjunctivitis	Allergen immunotherapy	RX
Short ragweed pollen allergen extract	Ragwizax^®^	Fast-dissolving tablet (Sublingual, Lyophilized)	ALK-Abelló (Horsholm, Denmark)	Allergic rhinitis; Conjunctivitis	Allergen immunotherapy	RX
Fentanyl citrate	Recivit^®^	Tablet (Sublingual)	Grünenthal GmbH (Aachen, Germany)	Breakthrough cancer pain	Opioid analgesic	RX
Sublivac	Sublivac^®^	Drop (Sublingual)	HAL Allergy Group (Leiden, The Netherlands)	Allergic rhinitis; Conjunctivitis	Allergen immunotherapy	RX
Buprenorphine + Naloxone	Zubsolv^®^	Tablet (Sublingual)	Orexo AB (Uppsala, Sweden)	Opioid dependence	Opioid agonist/antagonist	RX

**Table 3:** FDA-approved buccal and sublingual drug products.

Drug Name	Brand Name	Delivery Type	Company	Date of Approval	Bioavailability	Indication	Therapeutic Class	Status
Methyltestosterone	METANDREN	Tablet (Buccal/Sublingual)	Novartis Pharmaceuticals Corp. (East Hanover, NJ, USA)	Prior to 1 January 1982	~40% (buccal)	Male hypogonadism	Androgen	DISCN
Ergoloid mesylates	HYDERGINE	Tablet (Sublingual)	Sanofi Aventis US LLC. (Bridgewater, NJ, USA)	Prior to 1 January 1982	Not well quantified	Dementia, cognitive impairment	Ergot Alkaloid	DISCN
Isoproterenol hydrochloride	ISUPREL	Tablet (Sublingual/Rectal)	Organon USA Inc. (Jersey City, NJ, USA)	Prior to 1 January 1982	Not well quantified	Heart block, cardiac arrest, bronchospasm	Beta-Adrenergic Agonist	DISCN
Ergotamine tartrate	WIGRETTES	Tablet (Sublingual)	Biovail Laboratories Inc. (Bridgetown, Barbados)	29 July 1982	~2%	Migraine	Ergot Alkaloid	DISCN
Isosorbide dinitrate	ISORDIL	Tablet (Sublingual)	Pohl Boskamp (Hohenlockstedt, Germany)	29 July 1988	~25%	Angina	Nitrate Vasodilator	DISCN
Nitroglycerin	NITROLINGUAL	Aerosol, Metered (Sublingual)	Pohl Boskamp (Hohenlockstedt, Germany)	31 October 1985	~40%	Angina	Nitrate Vasodilator	DISCN
	NITROLINGUAL PUMPSPRAY	Spray, Metered (Sublingual)	Viatris Specialty LLC. (Morgantown, WV, USA)	10 January 1997	~40%			RX
	NITROSTAT	Tablet (Sublingual)	Haleon US Holdings LLC. (Warren, NJ, USA)	1 May 2000	~40%			RX
Nicotine polacrilex	NICORETTE	Chewing Gum (Buccal)	Noven Pharmaceuticals Inc. (Miami, FL, USA)	9 February 1996	~65–80% (buccal)	Smoking cessation	Nicotinic Agonist	OTC
Lidocaine	DENTIPATCH	Film, Extended Release (Buccal)	Indivior Inc. (North Chesterfield, VA, USA)	21 May 1996	~75% (localized buccal)	Local anesthesia	Local Anesthetic	DISCN
Buprenorphine hydrochloride	SUBUTEX	Tablet (Sublingual)	BioDelivery Sciences Intl. Inc. (Raleigh, NC, USA)	8 October 2002	~30–50%	Opioid dependence	Partial Opioid Agonist	DISCN
	BELBUCA	Film (Buccal)	Auxilium Pharmaceuticals LLC. (Malvern, PA, USA)	23 October 2015	~15% (buccal)			RX
Testosterone	STRIANT	Tablet, Extended Release (Buccal)	Cephalon LLC. (West Chester, PA, USA)	19 June 2003	~30% (buccal)	Male hypogonadism	Androgen	DISCN
Fentanyl citrate	FENTORA	Tablet (Buccal/Sublingual)	Adalvo Ltd. (San Gwann, Malta)	25 September 2006	~65% (buccal)	Breakthrough cancer pain	Opioid Analgesic	DISCN
	ONSOLIS	Film (Buccal)	Allergan Sales LLC. (Irvine, CA, USA)	16 July 2009	~65% (buccal)			DISCN
Asenapine maleate	SAPHRIS	Tablet (Sublingual)	Viatris Specialty LLC. (Morgantown, WV, USA)	13 August 2009	~35%	Schizophrenia, Bipolar Disorder	Atypical Antipsychotic	RX
Zolpidem tartrate	EDLUAR	Tablet (Sublingual)	Indivior Inc. (North Chesterfield, VA, USA)	13 March 2009	~70%	Insomnia	Sedative-Hypnotic	RX
Buprenorphine/ naloxone	SUBOXONE	Film (Buccal/Sublingual)	Galt Pharmaceuticals LLC. (Atlanta, GA, USA)	30 August 2010	~15% (buccal)	Opioid dependence	Opioid Agonist/Antagonist	RX
Miconazole	ORAVIG	Tablet (Buccal)	Ligand Pharmaceuticals Inc. (San Diego, CA, USA)	16 April 2010	~10% (buccal)	Oral candidiasis	Antifungal	RX
Acyclovir	SITAVIG	Tablet (Buccal)	Ferring Pharmaceuticals Inc. (Parsippany, NJ, USA)	12 April 2013	~30–50% (buccal)	Herpes labialis	Antiviral	RX
Desmopressin acetate	NOCDURNA	Tablet (Sublingual)	Sumitomo Pharma America Inc. (Brisbane, CA, USA)	21 June 2018	~0.25% (very low)	Nocturia	Vasopressin Analog	DISCN
Apomorphine HCl	KYNMOBI	Film (Sublingual)	BioXcel Therapeutics Inc. (New Haven, CT, USA)	21 May 2020	~17%	Parkinson’s episodes	Dopamine Agonist	DISCN
Dexmedetomidine HCl	IGALMI	Film (Buccal/Sublingual)	Novartis Pharmaceuticals Corp. (East Hanover, NJ, USA)	5 April 2022	Not publicly available	Acute agitation	Alpha-2 Adrenergic Agonist	RX

**Table 4:** Key advances in sublingual and buccal drug delivery (1950–2025).

Phase	Timeframe	Milestone/Innovation	Significance/Influence
Pioneering Efforts (1950–1982)	1950s	Buccal delivery of steroids	Bypassed hepatic first-pass; reduced injection dependency—foundational step for mucosal delivery.
	1970s	Propranolol sublingual absorption studies	Validated mucosal route for systemically active drugs; influenced later systemic delivery strategies.
	1970s	Bioelectric mucosal studies (aspirin, ethanol)	Revealed drug–mucosa interactions, guiding formulation strategies and penetration enhancer exploration.
Exploratory Growth (1983–1993)	1980s	Buccal/sublingual nitroglycerin (NTG)	Rapid relief for angina; inspired mucosal delivery in cardiovascular therapies.
	1980s	Nifedipine and Verapamil mucosal delivery	Enabled non-invasive emergency hypertension care; mimicked IV drug profiles.
	Late 1980s	Sublingual captopril	Accelerated therapeutic onset for hypertensive crises; bypassed GI absorption.
	1980s–1990s	Opioid exploration (morphine, fentanyl, buprenorphine)	Highlighted delivery challenges; spurred safer, more efficient transmucosal narcotic systems.
	Early 1990s	Peptide drug delivery studies	Exposed enzymatic barriers and low permeability; led to interest in nanoparticles and enhancers.
	Early 1990s	Transmucosal hormone systems (e.g., buccal testosterone)	Validated hormone delivery; emphasized role of pH, cosolvents, and formulation design.
	Early 1990s	Adhesive patches and controlled-release tablets	Sustained hormone delivery (6–12 h); improved compliance and set standards for future platforms.
Diversification & Discovery (1994–2009)	Late 1990s	Buprenorphine, fentanyl, oxycodone mucosal delivery	Expanded use for pain and opioid use disorder; validated mucosal route for potent narcotics.
	Late 1990s	Buccal testosterone and estrogens	Proven hormone absorption via mucosa; led to improved hormone replacement designs.
	Late 1990s	Mucoadhesive platforms (Cydot^®^, OTS^®^ tablets)	Enabled controlled release of peptides like GLP-1; emphasized comfort and patient adherence.
	Late 1990s–2000s	Peptide and protein drug trials (e.g., insulin, TRH)	Highlighted low permeability and enzymatic degradation; spurred interest in enhancers and nanoparticles.
	Early 2000s	Misoprostol sublingual vs buccal pharmacokinetics	Achieved faster onset and greater bioavailability via sublingual route; refined labor protocols.
	2000s	Liposomal buccal delivery systems	Encapsulated peptides; enhanced retention and enzymatic stability with mucoadhesive support.
	2000s	SLIT immunotherapy validation (e.g., Grazax^®^)	Efficacy confirmed by meta-analysis; enabled EU approval for grass pollen allergies.
	2000s	Human trials for buccal GLP-1, fentanyl lollipops	Demonstrated reliable absorption and clinical viability for mucosal delivery.
	2000s	Taste improvement and patient-centric formulations	Prioritized usability, mouthfeel, and adherence in buccal systems.
Innovation & Integration (2010–2025)	Early 2010s	Breakyl^®^ and Onsolis^®^ (BEMA^®^ buccal fentanyl films)	Enabled fast and discreet cancer pain therapy with bioerodible buccal films.
	Early 2010s	RapidMist™ buccal insulin spray (Generex Oral-lyn^®^)	Delivered insulin non-invasively; reduced HbA1c with high safety profile.
	2010s	Subsys^®^, Abstral^®^, Buccolam^®^, Intermezzo^®^ approvals	Validated transmucosal delivery for pain, seizures, insomnia, and pediatric emergencies.
	2010s	Suboxone^®^ buccal tablets and sublingual film (PharmFilm^®^)	Improved treatment for opioid dependence; minimized misuse risk.
	2010s	Sativex^®^ THC/CBD buccal spray	Approved for MS-related spasticity; expanded therapeutic cannabinoids in mucosal formats.
	2010s	Chemical and physical enhancers (DDAIP HCl, iontophoresis, etc.)	Increased mucosal permeability for various agents; unlocked new delivery targets.
	2010s	IntelliDrug intraoral device	Provided continuous electronic dosing; ideal for neurodegenerative conditions.
	2010s–2020s	Nanoparticle systems (PEG-coated, lipid/polymer)	Enhanced bioavailability and lymphatic uptake; enabled mucosal nano-formulations.
	2010s–2020s	Optimized misoprostol sublingual delivery	Superior pharmacokinetics improved labor induction protocols.
	2013–2017	Nanoparticle penetration studies (Teubl, Roblegg, Masek)	Size, charge, and formulation critically influenced mucosal transport.
	2020s	Ropivacaine liposomal gel in Phase I trials	Validated nanoparticle-based anesthetic via buccal route.
	2020s	Expanded drug indications (e.g., lorazepam, melatonin, etc.)	Demonstrated versatility across neurology, cardiology, psychiatry, and endocrinology.
	2020s	New dosage formats (films, lozenges, gels, sprays)	Boosted ease of use, comfort, and water-free administration.
	2020s	Mucoadhesive polymers (e.g., chitosan, hyaluronic acid)	Enhanced mucosal retention and drug absorption; improved formulation consistency.
	2020s	Vaccine delivery via buccal/sublingual routes	Achieved systemic and mucosal immunity; safer and pain-free alternatives to nasal/injection.
	2020s	VLPs, viral vectors, needle-free systems (e.g., MucoJet, microneedles)	Boosted antigen retention and immune activation; demonstrated clinical safety.
	2020s	Imaging advances (confocal, fluorescence, tissue clearing)	Enabled real-time visualization of antigen delivery and lymphatic activation.

## List of Abbreviations

ACE inhibitor	Angiotensin-Converting Enzyme Inhibitor
FDA	Food and Drug Administration
EMA	European Medicines Agency
WHO	World Health Organization
BN	Buccal Nitroglycerin
SLGTN	Sublingual Glyceryl Trinitrate
BGTN	Buccal Glyceryl Trinitrate
NTG	Nitroglycerin
TRH	Thyrotropin-Releasing Hormone
GLP-1	Glucagon-Like Peptide-1
FITC-dextran	Fluorescein Isothiocyanate-Dextran
ISMO	Isosorbide Mononitrate
APIs	Active Pharmaceutical Ingredients
Tmax	Time to Maximum Plasma Concentration
AUC	Area Under the Curve
LEV-INS	Liposome-Encapsulated Insulin
SLIT	Sublingual Immunotherapy
BEMA^®^	BioErodible MucoAdhesive
REMS	Risk Evaluation and Mitigation Strategy
PEG	Polyethylene Glycol
ALKS 5461	(No abbreviation; this is a drug code for a specific investigational medication)
VLPs	Virus-Like Particles
CTB	Cholera Toxin B
APC	Antigen-Presenting Cells
CYP3A4	Cytochrome P450 3A4
mRNA	Messenger Ribonucleic Acid
RX	Prescription only
OTC	Over the counter
DISCN	Discontinued
